# Socio-demographic, clinical and offense-related characteristics of forensic psychiatric inpatients in Hunan, China: a cross-sectional survey

**DOI:** 10.1186/s12888-022-04508-8

**Published:** 2023-01-18

**Authors:** Yu Gu, Huijuan Guo, Jiansong Zhou, Xiaoping Wang

**Affiliations:** Department of Psychiatry, National Clinical Research Center for Mental Disorders, The Second Xiangya Hospital of Central South University, Changsha, 410011 Hunan China

**Keywords:** Mentally ill offenders, Violent behavior, Forensic psychiatric hospital, China

## Abstract

**Background:**

There is still a lack of comprehensive research on the profile of patients in forensic mental health hospitals in China. This study aims to investigate the socio-demographic, clinical, and offense-related characteristics of mentally ill offenders in the Hunan Provincial Forensic Psychiatric Hospital in China.

**Methods:**

This study was conducted from November 1, 2018, to January 30, 2019. The data of socio-demographic, clinical, and offense-related characteristics of the patients were collected. The Brief Psychiatric Rating Scale (BPRS), the Modified Overt Aggression Scale (MOAS), and the Clinical Global Impression-Severity (CGI-S) scale were used to measure their psychiatric conditions.

**Results:**

A total of 461 participants were enrolled in this study. Among them, 86.3% were males and 56.8% were unmarried; the average age of them was 44.7 ± 10.1 years, and the mean years of education were 7.51 ± 3.3 years. Before their current offense, a total of 345 patients (74.8%) had sought medical help for their mental illnesses. While 303 (87.8%) of these patients were prescribed antipsychotics, 254 (73.6%) failed to take them regularly. Of all the inpatients, 90.5% were diagnosed with schizophrenia; 385 (83.5%) engaged in homicidal offenses, with 54.0% of the victims being their family members. In homicide cases, the relatives were more likely to be victims of female patients. The mean length of stay in the forensic hospital was 8.02 ± 4.74 years, and over 80.0% of the patients had been hospitalized for over 5 years.

**Conclusions:**

To our knowledge, this is the first study investigating the profile of forensic patients receiving compulsory treatments in a forensic psychiatric hospital in China. These results add to the world literature on the characteristics of forensic patients and can help identify common treatment and risk-related needs of this population.

**Supplementary Information:**

The online version contains supplementary material available at 10.1186/s12888-022-04508-8.

## Background

Mental illnesses, mainly presenting as cognitive, affective, and behavioral disorders, affect nearly 1 billion people worldwide [1]. It is generally known that mental illnesses bring a heavy disease burden to the patients themselves and the whole society [2], and some patients with serious mental illnesses (SMIs) may present with varying degrees of violence, ranging from verbal abuse to extreme violent behaviors such as intentional injury and homicide, under the influence of psychopathological factors. Previous studies have shown that people with mental disorders have a higher risk of violence than the general population, especially patients with schizophrenia [3, 4]. A number of studies in Western countries have shown that the incidence of violent behaviors in patients with schizophrenia was approximately 5 times higher than in the general population [5, 6]. A cross-sectional study conducted in China found that the average annual incidence of homicide in patients with schizophrenia was about 2.6 times higher than the rate of similar crimes in the general population nationwide [7]. The high incidence of violent crimes among patients with SMIs as well as the treatment and management of the patients have attracted great attention from researchers.

Previous studies have found that violent behaviors often recur among a small number of patients, especially those with first-episode or untreated mental disorders. A systematic review has shown that the risk of homicide is about 15 times higher during the first episode of psychosis as contrasted to later in the course of illness, with approximately one-third of such homicides occurring prior to the start of treatment [8]. Furthermore, Large et al. [9] found the increased risk of homicide in the first episode of psychosis was related to a longer duration of untreated psychosis (DUP), suggesting that violent offenses committed by patients could be prevented by the use of mental health services at an early stage. Patients with mental disorders at a high risk of violence not only cause safety issues to themselves and the public, but also are prone to adverse outcomes such as social withdrawal, self-injury and suicide, and recidivism. Therefore, in order to improve the patient’s condition and ensure social and public safety, it is necessary and beneficial to provide active treatment and management to patients with high risk of violence, especially those who have previously engaged in violent offenses [10].

At present, in most countries across the world, mentally ill offenders who are “not criminally responsible on account of mental disorder” (NCRMD) and still have the possibility of posing a risk to the public are usually sent to forensic psychiatric institutions for compulsory treatment. For example, in England and Wales, patients with major mental disorders who committed a crime and were judged to be “not guilty by reason of insanity (NGRI)” will be admitted to a secure psychiatric hospital [11]. Similarly, in the United States, NGRI patients are detained in psychiatric hospitals of medium to maximum security, and where they undergo periodic evaluations of their risk and mental status [12]. According to the Canadian Criminal Code, NCRMD patients need to be managed according to their grade of risk, the assessment of which is conducted by provincial Review Boards every year to decide whether the patient should continue hospitalization, be conditionally discharged or be unconditionally discharged [13, 14]. In China, NCRMD patients receive compulsory treatment in forensic psychiatric hospitals (Ankang Hospitals), which have similarities to the medium and high-security hospitals in the UK or maximum-security hospitals in the US [15]. These hospitals are supervised and funded by the government and operated by the Public Security Bureau. There are currently 25 forensic psychiatric hospitals housing over 7,000 patients in China [16].

At present, scholars have explored the characteristics of patients in forensic psychiatric hospitals in some countries. Previous study on forensic patients found that they often experienced lengthy stays in hospitals [17, 18], and they were also at a high risk of suicide [19]. Studies in China have similar findings [20, 21]. A retrospective study on the characteristics of NCRMD patients in the psychiatric hospitals of France found that the majority of the patients were diagnosed with psychotic disorder (61.8%), and young persons and males were overrepresented [22]. A study [23] in Germany partially supported these findings, in which 92.5% of patients were male with mean age of 39.1 years (*SD*: 11.5); while in this study the majority of diagnosis were substance use disorder (69.2%), while schizophrenic disorders were 24.2%, which may suggest that the characteristics of forensic patients may differ across jurisdiction.

There is still a lack of comprehensive research on the profile of patients in forensic mental hospitals in China. Compared with other countries in the world, China has different cultural backgrounds and legal systems, which may lead to different characteristics of Chinese forensic patients. At the same time, the subspecialty of forensic psychiatry in China was established relatively late, and needs further development [24]. In this context, the present study aims to investigate the socio-demographic, clinical, and offense-related characteristics of mentally ill offenders in the Hunan Provincial Forensic Psychiatric Hospital in China, to better understand the healthcare needs of such patients under a specific service model, and to inform future policymaking about the rehabilitation of forensic inpatients as well as studies of disposition outcomes in this field.

## Method

### Participants

This study was conducted from November 1, 2018, to January 30, 2019, in the Hunan Forensic Psychiatric Hospital, which is the only forensic psychiatric hospital in Hunan Province, China. Each of the investigators were trained by forensic psychiatrists and all questions in the interview had been discussed and confirmed by psychiatric experts. This study was approved by the Ethics Committees of the Second Xiangya Hospital and supported by Hunan Provincial Forensic Hospital. All participants signed the informed consent form, and were deemed competent to do so. We also obtained informed consent from the participant's parents or legal guardians to permit the authors to gather supplemental information from these individuals where necessary (e.g., regarding the patient's history of illness). The information of all participants was kept strictly confidential and only used for this research. All methods were performed in accordance with the relevant guidelines and regulations.

### Procedures

Before the start of the study, three forensic psychiatrists were trained on the use of assessment tools and consistency of data collection. An explanation of the purpose of this study was distributed by the investigators to all patients. After giving written informed consent, participants were asked to complete the standardized questionnaire with the assistance of a study investigator (when necessary). All participants were individually interviewed face-to-face in a private meeting room of the forensic psychiatric hospital, accompanied by a ward staff. Professors of our research group regularly supervised and supported the data collection process.

### Socio-demographic and clinical characteristics

A standard questionnaire was used to collect the participants’ socio-demographic and clinical characteristics, including gender, age, marital status, level of education, ethnicity, employment status, and living status prior to their current offense. The clinical characteristics included compliance to antipsychotic treatment before their current offense, length of the current hospital stay, current psychiatric diagnoses, current aggressive and violent behaviors, and level of illness severity.

The Brief Psychiatric Rating Scale (BPRS) was used to evaluate participants’ current psychiatric symptoms [25]. Each item in the BPRS was rated on a 7-point scale from “absence” to “extremely severe”, based on observation of the patient’s behaviors and speech during the face-to-face interview. The BPRS has shown good reliability and validity.

The Modified Overt Aggression Scale (MOAS) [26, 27] was used to assess the type and severity of any aggressive or violent behaviors committed by patients over the past month based on observation by medical staff prior to the interview. The MOAS is a 16-item checklist consisting of four categories, i.e., verbal aggression, aggression against properties, physical aggression against others, and auto aggression. Each category was rated on a 5-point scale (from 0 to 4), and the total score was calculated by summing the items falling in each category. The Chinese version of MOAS has demonstrated good reliability and validity [28].

The Clinical Global Impression Scale (CGI) is a tool used by experienced clinicians for the assessment of patients’ global function. It consists of two components, the CGI-Severity (CGI-S) for the assessment of illness severity, and the CGI-Improvement (CGI-I) for the assessment of changes during the current medical treatment. In the CGI-S, the severity of the disease was rated using a 7-point scale, from normal to extremely severe [29].

### Criminal information

Offense-related information, including the type of current offense, the number of the victim(s) in the homicide and intentional injury cases, and the relationship between the patient and victim(s), was obtained from the legal files of participants.

### Statistical analysis

Data were double-entered using Limesurvey software to minimize data entry errors and then exported to SPSS 23.0 for further analysis. For all socio-demographic, clinical, and offense-related variables, enumeration data were described using frequency and percentage, and measurement data were presented using averages and standard deviations. The characteristics of the offenses and the current diagnoses were compared between genders using a chi-square (χ^2^) test. For all analyses, the significance level was set at *p* < 0.05 (two-tailed).

## Results

### Socio-demographic characteristics

A total of 461 participants were enrolled in this study. Among them, 86.3% were males and 13.7% were females. Their mean age was 44.7 ± 10.1 years at the time of data collection (range: 21–80 years), and most of the participants (68.1%) were 31–50 years of age. The mean level of education was 7.51 ± 3.3 years, with most of the participants having a low level of education (primary school and lower: 48.1%; junior high school: 33.4%). Over half (56.8%) of the participants had never been married and 11.6% were divorced. The majority (95.0%) of participants were Han Chinese, and 88.1% lived in the rural area of China prior to hospital admission. Over half of the participants (55.1%) were unemployed and 76.8% lived with their families (including parents, siblings, spouses, and children) prior to their current offenses (Table 1).

**Table 1:** Socio-demographic characteristics

	Number of patients	Percentage (%)
**Gender**		
Male	398	86.3
Female	63	13.7
**Age (years)**		
≤ 30	35	7.6
31–40	122	26.5
41–50	192	41.6
50–60	78	16.9
≥ 60	34	7.4
**Level of education**		
Primary school and lower	222	48.1
Junior high school	154	33.4
High school	55	11.9
College and higher	3	0.7
Unavailable	27	5.9
**Marital status**		
Never married	262	56.8
Married	119	25.8
Cohabited	7	1.5
Widowed	8	1.8
Divorced	53	11.6
Separated	6	1.3
Unavailable	6	1.3
**Ethnicity**		
Han	438	95.0
Others	23	5.0
**Residence**		
Rural	406	88.1
Urban	54	11.7
Unavailable	1	0.2
**Living status**		
Alone	65	14.1
In a dormitory with others	7	1.5
Under supervision	1	0.2
With parents or siblings	248	53.8
With spouse or children	106	23.0
With remote relatives	1	0.2
Others	3	0.7
Unavailable	30	6.5
**Employment status**		
Unemployed	254	55.1
Employed	177	38.4
Unavailable	30	6.5

### Clinical characteristics

The analyses of past lifestyle and criminal history revealed that 186 participants (40.3%) had a history of alcohol use, 45 (9.7%) had a criminal history, 5 (1.1%) had a history of illegal substance use, and 299 (68.9%) were found to have engaged in aggressive behaviors before the current offense, based on self-report and available file information (Table 2).

**Table 2:** Lifestyle prior to hospitalization and clinical characteristics

	Number of cases	Percentage (%)
**History of smoking**		
Yes	216	46.9
No	215	46.6
Unavailable	30	6.5
**History of drinking**		
Yes	186	40.3
No	237	51.4
Unavailable	38	8.0
**History of illegal substance use**		
Yes	5	1.1
No	416	90.2
Unavailable	40	8.7
**History of aggressive behaviors**		
Yes	299	68.9
No	135	29.2
Unavailable	28	6.1
**Criminal history**		
Yes	45	9.7
No	392	84.8
Unavailable	25	5.9
**Family history of mental illness**		
Yes	54	11.7
No	367	79.6
Unavailable	40	8.7
**Seeking medical help for mental illness**		
Yes	345	74.8
No	116	25.2
**History of hospitalization in psychiatric hospitals**		
Yes	269	58.4
No	192	41.6
**Previous use of antipsychotics**		
Yes	303	65.7
No	158	44.3
**Irregular use of antipsychotics**		
Yes	254	55.1
No	207	54.9
**Current diagnosis**		
Schizophrenia	394	90.5
Major depressive disorder	15	3.4
Mental disorders due to epilepsy	10	2.3
Mental retardation	9	2.1
Bipolar disorder	8	1.8
Mental disorders due to substance use	5	1.1
Organic mental disorders	3	0.7
Schizoaffective disorders	2	0.5
Neurotic disorders	1	0.2
**Current mental status**		
BPRS (total scale score) ≤ 35 (29.63 ± 9.41)^a^	197	43.0
CGI-S = 1–3 (4,3) ^b^	204	51.7
MOAS (weighted total score) = 0 (0.23 ± 1.53)^a^	385	83.5
**Length of stay in forensic hospital (years)**		
≤ 5	130	28.2
6–10	224	48.6
11–15	83	18.0
≥ 16	21	4.6

^a^ Mean, SD

^b^ Median, quartiles

Before the current offense, a total of 345 participants (74.8%) had sought medical help for their mental illnesses, of whom 303 (87.8%) were given antipsychotics. Many patients in the sample (*n* = 269; 78.0%) had a history of psychiatric hospitalization prior to their current offenses, while 254 (73.6%) were not on regular antipsychotic treatment before the time of their offenses (Table 2).

A majority of the participants (*n* = 394, 90.5%) were diagnosed with schizophrenia, while only 15 (3.4%) were diagnosed with major depressive disorder. About half (43.0%) of patients obtained a BPRS total score of ≤ 35, and a similar proportion obtained CGI-S scores of ≤ 3 (51.7%). Most patients in this sample (*n* = 385; 83.5%) had a MOAS total score of 0.

By the time of enrolment, the mean length of stay in this forensic hospital was 8.02 (SD = 4.74) years, with the longest hospital stay being 37 years. Over 80.0% of patients had been hospitalized for over 5 years.

### Offense-related characteristics

Among the 461 inpatients, 385 (83.5%) had committed homicide, 51 (11.1%) had committed intentional injury, and 12 (2.6%) engaged in arson. Among the homicide cases, the number of deaths ranged from 0^1^ to 4, with approximately 10.0% of the cases resulting in ≥ 2 deaths. Most victims were known to the patients (relatives: 208, 54.0%; neighbors: 127, 33.0%), and only a relatively small number of victims were strangers to the patients (39, 10.1%). For cases of intentional injury, the number of victims ranged from 1 to 12, with 64.7% (33/51) involving one victim. Similar to homicide cases, the victims of intentional injury were mainly relatives (16, 31.4%) and neighbors (17, 33.3%) of the patients, and strangers only accounted for a small percentage (6, 11.8%). See Table 3 for more details.

**Table 3:** Offense-related characteristics

	Number of cases	Percentage (%)
**Type of current offense**		
Homicide	385	83.5
Intentional injury	51	11.1
Arson	12	2.6
Picking quarrels and provoking troubles	4	0.9
Unavailable	9	1.9
**Number of death(s)—homicide**		
0	12	3.1
1	333	86.5
2	32	8.3
3	5	1.3
≥ 4	3	0.8
**Number of victim(s)—intentional injury**		
1	33	64.7
2	7	13.7
3	5	9.8
≥ 4	6	11.7
**Patient’s relationship with the victim(s) – homicide**		
Relative	208	54.0
Neighbor	127	33.0
Stranger	39	10.1
Unavailable	12	3.1
**Patient’s relationship with the victim(s)—intentional injury**		
Relative	16	31.4
Neighbor	17	33.3
Stranger	6	11.8
Unavailable	12	23.5

Although relatives and neighbors accounted for the majority of victims in homicide cases, the relationship with the victim(s) differed between the genders of patients. Relatives (including family members, partners, and remote relatives) were more likely to be victims of female patients, while neighbors were more likely to be victims of male patients. Overall, relatives formed the most common victim group in homicides committed by males and females (as shown in Additional file 1).

## Discussion

To our knowledge, this is the first study to investigate the characteristics of inpatients in a forensic psychiatric hospital in China. In this study, we found that most of the patients were male, unmarried, unemployed, and had a lower level of education, and most of them were diagnosed with schizophrenia and had been hospitalized for a lengthy period. The main types of violence were homicide and intentional injury, with relatives being the most common victims and strangers being infrequently represented. Gender differences were also found in cases of homicide, with men more likely to offend against neighbors and women more likely to offend against relatives. These findings may have implications for the treatment and management of inpatients in forensic psychiatric institutions as well as individuals with mental disorders who are likely to engage in violent behaviors.

The current sample of forensic inpatients were predominately males, which is consistent with the findings of other studies internationally [30, 31]. We also found that most of the patients had experienced negative life events before the offense, such as having an unmarried status, unemployment, and low levels of education, and which is consistent with earlier studies [32–35]. In the present study, the majority of patients lived with their families/relatives before the offense; this might be related to the patient’s impairment in social function due to mental illness, which hindered them from living and working independently. This dependence, as well as the impaired social function, might also be a reason for the over-representativeness of family members/relatives among victims.

We also found that more than 70.0% of the participants had sought prior medical attention due to mental illnesses. Among them, over 80.0% were prescribed antipsychotics and 78.0% had been admitted to psychiatric hospitals; however, 73.6% failed to take antipsychotics regularly. It can be seen that before their offenses, a large number of patients were non-compliant with their treatment, which might have led to poor control of their diseases. We speculated that the reasons for the poor compliance might be that 1) patients and their families lacked knowledge about their mental illnesses and the necessity of long-term treatment; 2) patients and their families experienced stigma towards mental illnesses, which resulted in the refusal of long-term treatment; and 3) the patients might not be able to maintain their jobs due to impaired function, leading to reduced income as well as the ability to afford the medications they needed [36]. Based on our findings and speculation, targeted interventions, such as educational programs on the nature of mental illnesses and the safety of family and caregivers, can be developed to improve the public understanding of mental diseases and reduce the stigma surrounding these patients. Our findings may also raise awareness of the importance of standardized treatment of mental illnesses.

The present study showed that most of the patients were diagnosed with schizophrenia (more than 90.0%). The percentage is far higher than that of patients with other mental disorders, which is in line with previous studies from forensic settings in other countries [37, 38]. Generally, patients with severe schizophrenia often have a long course of the disease, poor self-care ability, and poor social function, and some patients may even engage in violent behaviors under the influence of their psychotic symptoms. Previous studies revealed that patients with schizophrenia were often overrepresented, as compared with patients with other mental disorders, among individuals engaging in extremely violent behaviors such as intentional injury and homicide [4, 39]. In addition, our findings showed that only 1.1% of the patients (5 cases) had a history of illegal substance use, which is far lower than those reported in other countries [40]. A possible reason is that illegal substances are strictly prohibited and controlled in China, as compared to many Western countries.

Concerning offense-related characteristics, we found that patients who engaged in homicides and intentional injury accounted for a high proportion of the sample. The Hunan Provincial Forensic Psychiatric Hospital is the only institution in Hunan Province that is designated to detain NCRMD patients, and so it is not surprising that this level of care is mainly reserved for patients who have committed serious violent crimes. Moreover, we also found that family members formed the biggest group of victims in homicide (54.0%) and injury cases (31.4%), followed by neighbors (33.0% and 33.3%, respectively), strangers (10.1% and 11.8%, respectively), which is again consistent with research from other countries. For example, Golenkov et al. [41] reported that most victims of homicide committed by persons with schizophrenia in Russia were familial (57.0%) or neighbors (39.5%), while the proportion of stranger victims was 3.5% in this 40-year data-set sample. Similarly, in a Belgian study of NGRI acquittees [42], 59.8% of homicide victims were family members and 15.5% were strangers. This pattern of findings might be partially attributed to the patient’s lack of ability to maintain their jobs due to the influence of the disease, leading to more time staying within their neighborhoods and at home with their families [43].

Furthermore, the deaths or injuries caused by patients may be especially likely to cause panic and fear in the community or village if this is where the offenses are primarily being committed. The above problems again highlight the importance of public education about mental health and reducing the stigma of mentally ill patients and their families. To ensure the safety of the public, it may also be necessary to improve community mental health services which encompasses more proficient risk assessment and management, trying to provide more efficient help and support for persons with mental disorders who are at a high risk of engaging in violence, before they commit crimes in the first place.

Through the analysis of the offense characteristics and diagnoses of the patient group, we found few gender differences in the nature of the crime, number of victims in homicide and intentional injury cases, and current diagnoses. However, gender differences were observed in the relationship with victims of homicides. In the present study, female inpatients were more likely than males to harm their family members in cases resulting in deaths. This finding may indicate the need for gender-specific interventions. Gender differences should also be taken into account in the formulation of rehabilitation programs and post-discharge policies of forensic mental health services.

Our findings showed that about 20.0% of patients had been hospitalized for more than 10 years, with a maximum hospital stay being 37 years. Long hospital stays are also found to be common in judicial psychiatric hospitals in other countries and regions [18, 44, 45]. Possible factors of the long hospital stay of forensic patients in China include the lack of laws and regulations on the length of hospitalization for NCRMD patients, the persistent psychotic symptoms of some patients [46], and the lack of family and social support [47]. Patients with serious mental illnesses, little family support, or absence of guardianship (e.g., guardians became victims) are less likely to meet the criteria for discharge, resulting in a longer hospital stay. Studies have shown that longer stay in forensic psychiatric hospitals may also cause psychological distress (e.g., depression) and negative clinical outcomes [20], which necessitates regular psychological interventions for forensic patients. Additional post-discharge programs should also be provided for those who have little family and social support.

Despite the strengths, there are still limitations to this study. This study on the profile of forensic psychiatric patients is cross-sectional, making it difficult to draw causal inferences as to the underlying factors responsible for the commission of the current offenses or subsequent forensic admission. Hunan Province Forensic Psychiatric Hospital is the only forensic facility admitting mentally ill offenders in Hunan Province; thus, it is still unknown whether the demographic, clinical, and offense-related profiles of these patients are generalizable to other areas of China. In the future, longitudinal and larger-scale studies on the profile of mentally ill offenders are warranted to improve our understanding of the features of such populations across this large country.

## Conclusions

To our knowledge, this is the first study investigating the profile of patients receiving compulsory treatment in a forensic psychiatric hospital in China. This study found that most of the patients were male and unmarried, and had a low level of education; most of them were diagnosed with schizophrenia and hospitalized for a lengthy period. The main types of crimes were homicide and intentional injury, with family members accounting for over half of the victims. These findings begin to help identify common treatment and risk-related needs of forensic patients in China.

## Supplementary Information

**Additional file 1:** Gender differences. 

## Data Availability

The datasets used and/or analyzed in the present study are available from the corresponding author upon reasonable request.

## Abbreviations

SMI: Serious mental illness
DUP: Duration of untreated psychosis
NGRI: Not guilty by reason of insanity
NCRMD: Not criminally responsible on account of mental disorder
BPRS: The Brief Psychiatric Rating Scale
MOAS: The Modified Overt Aggression Scale
CGI: Clinical Global Impression Scale
CGI-S: Clinical Global Impression Scale of Severity
CGI-I: Clinical Global Impression Scale of Improvement

## Footnotes

1 According to the Chinese Criminal Law and judicial interpretation, intentional homicide refers to the act of intentionally and illegally depriving others of their lives, which is a crime of violating citizens' personal and democratic rights. The crime of intentional homicide is a behavioral crime. As long as the actor commits the act of intentional homicide, it constitutes the crime of intentional homicide. Regardless of whether the victim is dead or not, and regardless of whether the homicide is at any stage of preparation, attempt, or suspension of an intentional crime, it constitutes a crime. This means that the number of deaths of the victims does not affect the conviction of intentional homicide. Therefore, someone would have an offense of homicide with no deaths.
