# Supplementary material for: Socio-demographic, clinical and offense-related characteristics of forensic psychiatric inpatients in Hunan, China: a cross-sectional survey
Source: BMC Psychiatry. 2023 Jan 18;23:48. doi: 10.1186/s12888-022-04508-8 (PMC9847096; doi:10.1186/s12888-022-04508-8)
Supplement: Supplementary file 1 — Additional file 1: Gender differences. [file 12888_2022_4508_MOESM1_ESM.pdf]

Additional table 1: The gender differences in the criminological characteristics and diagnoses of the mentally ill offenders

| | Total (N=461) | | Male (N=398) | | Female (N=63) | | |
|---|---|---|---|---|---|---|---|
| | N | % | N | % | N | % | $\chi^2$ |
| **Type of current offense** | | | | | | | 2.363 |
| Homicide | 385 | 83.5 | 329 | 82.7 | 56 | 88.9 | |
| Serious bodily injuries | 51 | 11.1 | 44 | 11.1 | 7 | 11.1 | |
| Arson | 12 | 2.6 | 12 | 3.0 | 0 | 0.0 | |
| Picking quarrels and provoking troubles | 4 | 0.9 | 4 | 1.0 | 0 | 0.0 | |
| Unavailable | 9 | 1.9 | 9 | 2.3 | 0 | 0.0 | |
| **Number of death(s) - homicide** | | | | | | | 0.982 |
| 0 | 12 | 3.1 | 10 | 3.0 | 2 | 3.6 | |
| 1 | 333 | 86.5 | 285 | 86.6 | 48 | 85.7 | |
| 2 | 32 | 8.3 | 27 | 8.2 | 5 | 8.9 | |
| 3 | 5 | 1.3 | 4 | 1.2 | 1 | 1.8 | |
| ≥4 | 3 | 0.8 | 3 | 0.9 | 0 | 0.0 | |
| **Number of victim(s) - intentional injury** | | | | | | | 6.017 |
| 1 | 33 | 64.7 | 30 | 68.2 | 3 | 42.9 | |
| 2 | 7 | 13.7 | 6 | 13.6 | 1 | 14.3 | |
| 3 | 5 | 9.8 | 5 | 11.4 | 0 | 0.0 | |
| ≥4 | 6 | 11.7 | 3 | 6.8 | 3 | 42.9 | |
| **Patient's relationship with victim(s) - homicide** | | | | | | | 8.881* |
| Relative | 208 | 54.0 | 168 | 51.1 | 40 | 71.4 | 7.990** |
| Neighbor | 127 | 33.0 | 116 | 35.3 | 10 | 17.9 | 6.582* |
| Stranger | 39 | 10.1 | 35 | 10.6 | 4 | 7.1 | 0.642 |
| Unavailable | 12 | 3.1 | 10 | 3.0 | 2 | 3.6 | 0.045 |
| **Patient's relationship with victim(s) - intentional injury** | | | | | | | 5.095 |
| Relative | 16 | 31.4 | 12 | 27.3 | 4 | 57.1 | |
| Neighbor | 17 | 33.3 | 17 | 38.6 | 0 | 0.0 | |
| Stranger | 6 | 11.8 | 5 | 11.4 | 1 | 14.3 | |
| Unavailable | 12 | 23.5 | 10 | 22.7 | 2 | 28.6 | |
| **Current diagnosis** | | | | | | | 2.375 |

| | | | | | | |
|---|---|---|---|---|---|---|
| Schizophrenia | 394 | 85.5% | 345 | 86.7 | 50 | 79.4 |
| Others | 67 | 14.5% | 53 | 13.3 | 13 | 20.6 |

*$p$ <0.05, **$p$ <0.01
